# 1-Benzyl-5-meth­oxy-2′,3-dimethyl-4,6-dioxa-2-aza­spiro­[bicyclo­[3.2.0]hept-2-ene-7,4′-isoquinoline]-1′,3′(2′*H*,4′*H*)-dione

**DOI:** 10.1107/S160053681101600X

**Published:** 2011-05-07

**Authors:** Hoong-Kun Fun, Ching Kheng Quah, Chengmei Huang, Haitao Yu

**Affiliations:** a X-ray Crystallography Unit, School of Physics, Universiti Sains Malaysia, 11800 USM, Penang, Malaysia; b School of Chemistry and Chemical Engineering, Nanjing University, Nanjing, 210093, People’s Republic of China

## Abstract

In the isoquinoline ring system of the title mol­ecule, C_22_H_20_N_2_O_5_, the *N*-heterocyclic ring is in a half-boat conformation. The dioxa-2-aza­spiro ring is essentially planar [maximum deviation = 0.026 (1) Å] and forms dihedral angles of 22.53 (5) and 64.46 (5)° with the benzene and phenyl rings, respectively. The mol­ecular structure is stabilized by a weak intra­molecular C—H⋯O hydrogen bond, which generates an *S*(7) ring motif. In the crystal, mol­ecules are linked *via* weak inter­molecular C—H⋯O and C—H⋯N hydrogen bonds into layers parallel to (102).

## Related literature

For general background to and the potential biological activity of the title compound, see: Du *et al.* (2008 ▶); Chen *et al.* (2006 ▶); Yu *et al.* (2010 ▶); Harris *et al.* (2005 ▶); Zhang *et al.* (2004 ▶); Wang *et al.* (2010 ▶); Huang *et al.* (2011 ▶). For the stability of the temperature controller used in the data collection, see: Cosier & Glazer (1986 ▶). For standard bond-length data, see: Allen *et al.* (1987 ▶). For ring conformations, see: Cremer & Pople (1975 ▶). For hydrogen-bond motifs, see: Bernstein *et al.* (1995 ▶). For related structures, see: Fun *et al.* (2011*a* ▶,*b* ▶).
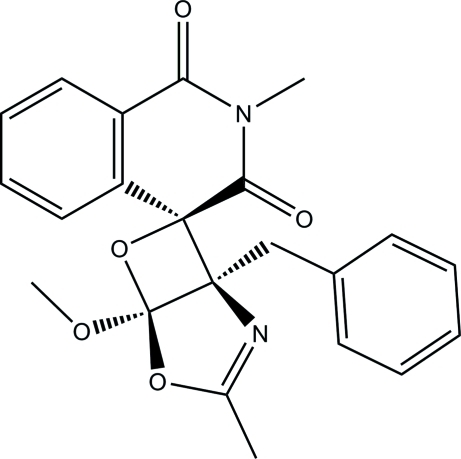


## Experimental

### 

#### Crystal data

C_22_H_20_N_2_O_5_
*M* *_r_* = 392.40
Monoclinic, 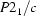

*a* = 9.7261 (2) Å
*b* = 12.4444 (2) Å
*c* = 15.8413 (3) Å
β = 108.884 (1)°
*V* = 1814.16 (6) Å^3^
*Z* = 4
Mo *K*α radiation
μ = 0.10 mm^−1^
*T* = 100 K
0.71 × 0.44 × 0.25 mm

#### Data collection

Bruker SMART APEXII CCD area-detector diffractometer
Absorption correction: multi-scan (*SADABS*; Bruker, 2009 ▶) *T*_min_ = 0.931, *T*_max_ = 0.975
21150 measured reflections
5368 independent reflections
4883 reflections with *I* > 2σ(*I*)
*R*_int_ = 0.019

#### Refinement

*R*[*F* ^2^ > 2σ(*F* ^2^)] = 0.037
*wR*(*F* ^2^) = 0.100
*S* = 1.02
5368 reflections
265 parameters
H-atom parameters constrained
Δρ_max_ = 0.51 e Å^−3^
Δρ_min_ = −0.27 e Å^−3^

### 

Data collection: *APEX2* (Bruker, 2009 ▶); cell refinement: *SAINT* (Bruker, 2009 ▶); data reduction: *SAINT*; program(s) used to solve structure: *SHELXTL* (Sheldrick, 2008 ▶); program(s) used to refine structure: *SHELXTL*; molecular graphics: *SHELXTL*; software used to prepare material for publication: *SHELXTL* and *PLATON* (Spek, 2009 ▶).

## Supplementary Material

Crystal structure: contains datablocks global, I. DOI: 10.1107/S160053681101600X/lh5240sup1.cif

Structure factors: contains datablocks I. DOI: 10.1107/S160053681101600X/lh5240Isup2.hkl

Supplementary material file. DOI: 10.1107/S160053681101600X/lh5240Isup3.cml

Additional supplementary materials:  crystallographic information; 3D view; checkCIF report

## Figures and Tables

**Table 1:** Hydrogen-bond geometry (Å, °)

*D*—H⋯*A*	*D*—H	H⋯*A*	*D*⋯*A*	*D*—H⋯*A*
C15—H15*A*⋯O5	0.93	2.51	3.3026 (12)	143
C20—H20*C*⋯O2^i^	0.96	2.49	3.4424 (13)	174
C21—H21*B*⋯N2^ii^	0.96	2.52	3.4018 (12)	152
C22—H22*C*⋯O2^ii^	0.96	2.50	3.3854 (12)	154

Symmetry codes: (i) 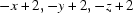; (ii) 
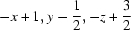
.

**Fig. 1.**
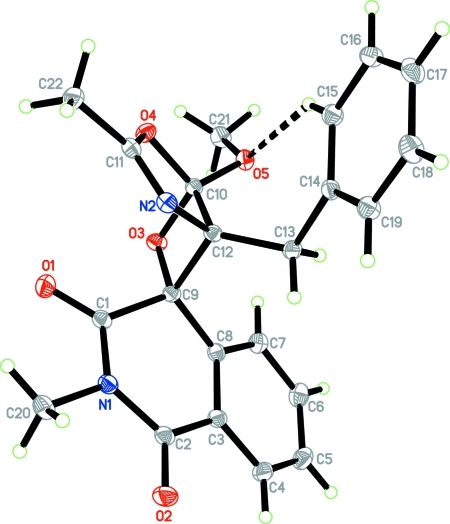
The molecular structure of the title compound showing 50% probability displacement ellipsoids for non-H atoms. A weak intramolecular hydrogen bond is shown as a dashed line.

**Fig. 2.**
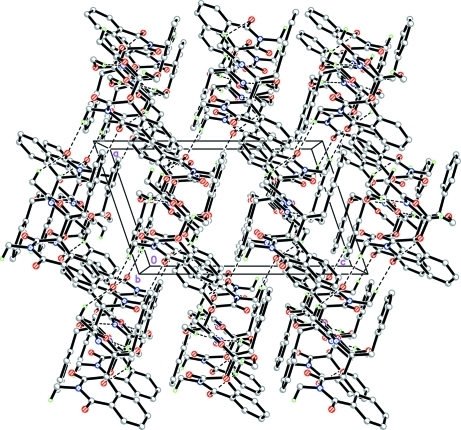
The crystal structure of the title compound, viewed along the b axis. H atoms not involved in hydrogen bonds (dashed lines) have been omitted for clarity.

## supplementary crystallographic information

### Comment

Isoquinoline-1,3,4-trione derivatives were reported to be a type of small molecular inhibitor against caspase-3 which can promote apoptosis of the cells (Du *et al.*, 2008; Chen *et al.*, 2006). Compounds containing an oxazole moiety have been found to inhibit the activity of malignant tumors (Harris *et al.*, 2005). Since many natural products especially the alkaloids containing isoquinoline or oxazole ring are bioactive, there has been intense interest in building frameworks containing isoquinoline moieties with an oxazole group (Yu *et al.*, 2010; Zhang *et al.*, 2004; Wang *et al.*, 2010). The title compound was derived from photocycloaddition of isoquinoline-1,3,4-trione and oxazole (Huang *et al.*, 2011). Since it may have a potential use in biochemical and pharmaceutical fields, we report in this paper the crystal structure of the title compound with a relative configuration of (1S^*^, 4'S^*^, 5R^*^).

In the title racemic compound, Fig. 1, atoms C9, C10 and C12 are the stereo centers. The isoquinoline ring system (N1/C1-C9) is not completely planar, the *N*-heterocyclic ring (N1/C1-C3/C8/C9) being distorted towards a half-boat conformation with atom C9 deviating by 0.222 (1) Å from the mean plane through the remaining atoms, puckering parameters (Cremer & Pople, 1975) Q = 0.329 (1) Å, Θ = 62.51 (17)° and φ = 104.40 (18)°. The dioxa-2-azaspiro ring (N2/O4/C10-C12) is essentially planar [maximum deviation of 0.026 (1) Å at atoms O4 and C10] and it inclines at dihedral angles of 22.53 (5) and 64.46 (5)° with the benzene and phenyl rings (C3-C8 and C14-C19), respectively. Bond lengths (Allen *et al.*, 1987) and angles are within normal ranges and comparable to related structures (Fun *et al.*, 2011*a*, *b*). The molecular structure is stabilized by a weak intramolecular C15–H15A···O5 hydrogen bond (Table 1) which generates a *S*(7) ring motif (Fig. 1, Bernstein *et al.*, 1995).

In the crystal structure, Fig. 2, molecules are linked *via* intermolecular C20–H20C···O2^i^, C21–H21B···N2^ii^ and C22–H22C···O2^ii^ hydrogen bonds (see Table 1 for symmetry codes) into two-dimensional planes parallel to (102).

### Experimental

The title compound was the main product from the photoreaction between isoquinoline-1,3,4-trione and 4-benzyl-5-methoxy-2-methyloxazole. The compound was purified by flash column chromatography with ethyl acetate/petroleum ether (1:4) as eluents. X-ray quality crystals of the title compound was obtained from slow evaporation of an acetone and petroleum ether solution (1:5) (*m.p.* 463-465 K).

### Refinement

All H atoms were positioned geometrically and refined using a riding model with C–H = 0.93 - 0.97 Å and *U*_iso_(H) = 1.2 or 1.5 *U*_eq_(C). A rotating-group model was applied for the methyl groups. The highest residual electron density peak is located at 0.75 Å from C1 and the deepest hole is located at 0.59 Å from C10.

### Crystal data

**Table:**

C_22_H_20_N_2_O_5_	*F*(000) = 824
*M**_r_* = 392.40	*D*_x_ = 1.437 Mg m^−^^3^
Monoclinic, *P*2_1_/*c*	Mo *K*α radiation, λ = 0.71073 Å
Hall symbol: -P 2ybc	Cell parameters from 9898 reflections
*a* = 9.7261 (2) Å	θ = 2.7–30.2°
*b* = 12.4444 (2) Å	µ = 0.10 mm^−^^1^
*c* = 15.8413 (3) Å	*T* = 100 K
β = 108.884 (1)°	Block, colourless
*V* = 1814.16 (6) Å^3^	0.71 × 0.44 × 0.25 mm
*Z* = 4	

### Data collection

**Table:**

Bruker SMART APEXII CCD area-detector diffractometer	5368 independent reflections
Radiation source: fine-focus sealed tube	4883 reflections with *I* > 2σ(*I*)
graphite	*R*_int_ = 0.019
φ and ω scans	θ_max_ = 30.2°, θ_min_ = 2.1°
Absorption correction: multi-scan (*SADABS*; Bruker, 2009)	*h* = −13→13
*T*_min_ = 0.931, *T*_max_ = 0.975	*k* = −17→17
21150 measured reflections	*l* = −15→22

### Refinement

**Table:**

Refinement on *F*^2^	Primary atom site location: structure-invariant direct methods
Least-squares matrix: full	Secondary atom site location: difference Fourier map
*R*[*F*^2^ > 2σ(*F*^2^)] = 0.037	Hydrogen site location: inferred from neighbouring sites
*wR*(*F*^2^) = 0.100	H-atom parameters constrained
*S* = 1.02	*w* = 1/[σ^2^(*F*_o_^2^) + (0.0545*P*)^2^ + 0.7208*P*] where *P* = (*F*_o_^2^ + 2*F*_c_^2^)/3
5368 reflections	(Δ/σ)_max_ = 0.001
265 parameters	Δρ_max_ = 0.51 e Å^−^^3^
0 restraints	Δρ_min_ = −0.27 e Å^−^^3^

### Special details

**Table:**

Experimental. The crystal was placed in the cold stream of an Oxford Cryosystems Cobra open-flow nitrogen cryostat (Cosier & Glazer, 1986) operating at 100.0 (1) K.
Geometry. All esds (except the esd in the dihedral angle between two l.s. planes) are estimated using the full covariance matrix. The cell esds are taken into account individually in the estimation of esds in distances, angles and torsion angles; correlations between esds in cell parameters are only used when they are defined by crystal symmetry. An approximate (isotropic) treatment of cell esds is used for estimating esds involving l.s. planes.
Refinement. Refinement of F^2^ against ALL reflections. The weighted R-factor wR and goodness of fit S are based on F^2^, conventional R-factors R are based on F, with F set to zero for negative F^2^. The threshold expression of F^2^ > 2sigma(F^2^) is used only for calculating R-factors(gt) etc. and is not relevant to the choice of reflections for refinement. R-factors based on F^2^ are statistically about twice as large as those based on F, and R- factors based on ALL data will be even larger.

### Fractional atomic coordinates and isotropic or equivalent isotropic displacement parameters (Å^2^)

**Table:**

	*x*	*y*	*z*	*U*_iso_*/*U*_eq_	
O1	0.70635 (8)	0.71051 (6)	0.91416 (5)	0.01856 (15)	
O2	0.90622 (8)	1.02544 (6)	0.87008 (5)	0.01959 (15)	
O3	0.71792 (7)	0.61939 (5)	0.75908 (4)	0.01336 (13)	
O4	0.46875 (7)	0.58791 (5)	0.73113 (4)	0.01357 (13)	
O5	0.56352 (7)	0.57911 (5)	0.61557 (4)	0.01337 (13)	
N1	0.78670 (8)	0.87467 (6)	0.88611 (5)	0.01347 (15)	
N2	0.46589 (8)	0.77013 (6)	0.74800 (5)	0.01303 (15)	
C1	0.74250 (9)	0.76872 (7)	0.86397 (6)	0.01282 (16)	
C2	0.86611 (10)	0.93487 (7)	0.84373 (6)	0.01376 (16)	
C3	0.90674 (9)	0.88137 (7)	0.77166 (6)	0.01332 (16)	
C4	1.00910 (10)	0.93045 (8)	0.73946 (6)	0.01692 (18)	
H4A	1.0434	0.9988	0.7590	0.020*	
C5	1.05954 (10)	0.87726 (8)	0.67839 (7)	0.01917 (19)	
H5A	1.1287	0.9094	0.6576	0.023*	
C6	1.00631 (11)	0.77555 (8)	0.64832 (7)	0.01886 (19)	
H6A	1.0416	0.7393	0.6083	0.023*	
C7	0.90070 (10)	0.72769 (8)	0.67776 (6)	0.01571 (17)	
H7A	0.8635	0.6607	0.6560	0.019*	
C8	0.85086 (9)	0.78033 (7)	0.73984 (6)	0.01255 (16)	
C9	0.73190 (9)	0.73502 (7)	0.76955 (6)	0.01148 (15)	
C10	0.57599 (9)	0.62694 (7)	0.69477 (6)	0.01140 (16)	
C11	0.41832 (10)	0.67863 (7)	0.76214 (6)	0.01311 (16)	
C12	0.57073 (9)	0.75055 (7)	0.70176 (6)	0.01121 (15)	
C13	0.55210 (10)	0.82504 (7)	0.62266 (6)	0.01359 (16)	
H13A	0.5951	0.8941	0.6447	0.016*	
H13B	0.6053	0.7954	0.5858	0.016*	
C14	0.39571 (10)	0.84262 (7)	0.56521 (6)	0.01299 (16)	
C15	0.30401 (10)	0.75654 (8)	0.52783 (6)	0.01561 (17)	
H15A	0.3384	0.6865	0.5397	0.019*	
C16	0.16165 (10)	0.77436 (8)	0.47305 (6)	0.01777 (18)	
H16A	0.1016	0.7163	0.4488	0.021*	
C17	0.10889 (10)	0.87872 (9)	0.45445 (6)	0.01908 (19)	
H17A	0.0142	0.8906	0.4173	0.023*	
C18	0.19865 (11)	0.96482 (8)	0.49175 (7)	0.01907 (19)	
H18A	0.1639	1.0347	0.4798	0.023*	
C19	0.34088 (10)	0.94687 (8)	0.54716 (6)	0.01628 (17)	
H19A	0.3999	1.0051	0.5724	0.020*	
C20	0.76802 (11)	0.92219 (8)	0.96661 (6)	0.01787 (18)	
H20A	0.7099	0.8752	0.9893	0.027*	
H20B	0.7205	0.9905	0.9520	0.027*	
H20C	0.8614	0.9318	1.0111	0.027*	
C21	0.60558 (10)	0.46687 (7)	0.62301 (6)	0.01648 (17)	
H21A	0.5714	0.4339	0.5652	0.025*	
H21B	0.5637	0.4310	0.6625	0.025*	
H21C	0.7095	0.4615	0.6463	0.025*	
C22	0.31256 (10)	0.65843 (8)	0.80979 (6)	0.01721 (18)	
H22A	0.2786	0.7257	0.8251	0.026*	
H22B	0.3587	0.6182	0.8632	0.026*	
H22C	0.2318	0.6182	0.7720	0.026*	

### Atomic displacement parameters (Å^2^)

**Table:**

	*U*^11^	*U*^22^	*U*^33^	*U*^12^	*U*^13^	*U*^23^
O1	0.0235 (3)	0.0169 (3)	0.0153 (3)	−0.0026 (3)	0.0065 (3)	0.0021 (3)
O2	0.0232 (3)	0.0136 (3)	0.0192 (3)	−0.0043 (3)	0.0031 (3)	−0.0019 (3)
O3	0.0134 (3)	0.0090 (3)	0.0152 (3)	0.0000 (2)	0.0012 (2)	0.0001 (2)
O4	0.0156 (3)	0.0110 (3)	0.0158 (3)	−0.0014 (2)	0.0074 (2)	−0.0007 (2)
O5	0.0181 (3)	0.0099 (3)	0.0120 (3)	0.0012 (2)	0.0047 (2)	−0.0010 (2)
N1	0.0148 (3)	0.0127 (3)	0.0119 (3)	−0.0008 (3)	0.0030 (3)	−0.0011 (3)
N2	0.0123 (3)	0.0137 (3)	0.0127 (3)	0.0009 (3)	0.0035 (3)	−0.0012 (3)
C1	0.0118 (4)	0.0126 (4)	0.0122 (4)	0.0006 (3)	0.0013 (3)	0.0000 (3)
C2	0.0127 (4)	0.0129 (4)	0.0131 (4)	−0.0003 (3)	0.0004 (3)	0.0012 (3)
C3	0.0118 (4)	0.0133 (4)	0.0131 (4)	0.0000 (3)	0.0016 (3)	0.0015 (3)
C4	0.0141 (4)	0.0168 (4)	0.0180 (4)	−0.0023 (3)	0.0026 (3)	0.0034 (3)
C5	0.0149 (4)	0.0227 (5)	0.0204 (4)	0.0003 (3)	0.0064 (3)	0.0064 (4)
C6	0.0185 (4)	0.0211 (5)	0.0191 (4)	0.0040 (3)	0.0089 (4)	0.0034 (3)
C7	0.0163 (4)	0.0147 (4)	0.0162 (4)	0.0019 (3)	0.0053 (3)	0.0011 (3)
C8	0.0113 (4)	0.0127 (4)	0.0123 (4)	0.0008 (3)	0.0018 (3)	0.0019 (3)
C9	0.0125 (4)	0.0088 (3)	0.0120 (4)	−0.0001 (3)	0.0025 (3)	0.0002 (3)
C10	0.0121 (4)	0.0103 (3)	0.0114 (4)	−0.0003 (3)	0.0032 (3)	0.0002 (3)
C11	0.0134 (4)	0.0142 (4)	0.0110 (4)	0.0005 (3)	0.0029 (3)	−0.0016 (3)
C12	0.0115 (3)	0.0098 (3)	0.0114 (4)	−0.0001 (3)	0.0025 (3)	−0.0008 (3)
C13	0.0126 (4)	0.0121 (4)	0.0145 (4)	−0.0002 (3)	0.0023 (3)	0.0027 (3)
C14	0.0133 (4)	0.0137 (4)	0.0114 (4)	0.0007 (3)	0.0032 (3)	0.0012 (3)
C15	0.0160 (4)	0.0146 (4)	0.0154 (4)	−0.0001 (3)	0.0040 (3)	0.0004 (3)
C16	0.0148 (4)	0.0216 (5)	0.0160 (4)	−0.0033 (3)	0.0037 (3)	−0.0014 (3)
C17	0.0131 (4)	0.0270 (5)	0.0157 (4)	0.0030 (3)	0.0026 (3)	0.0014 (4)
C18	0.0181 (4)	0.0185 (4)	0.0188 (4)	0.0063 (3)	0.0035 (3)	0.0021 (3)
C19	0.0166 (4)	0.0140 (4)	0.0164 (4)	0.0011 (3)	0.0029 (3)	0.0002 (3)
C20	0.0207 (4)	0.0191 (4)	0.0135 (4)	−0.0005 (3)	0.0052 (3)	−0.0037 (3)
C21	0.0208 (4)	0.0107 (4)	0.0170 (4)	0.0020 (3)	0.0049 (3)	−0.0016 (3)
C22	0.0175 (4)	0.0196 (4)	0.0167 (4)	−0.0016 (3)	0.0085 (3)	−0.0009 (3)

### Geometric parameters (Å, °)

**Table:**

O1—C1	1.2089 (11)	C10—C12	1.5443 (12)
O2—C2	1.2212 (11)	C11—C22	1.4810 (12)
O3—C10	1.4290 (10)	C12—C13	1.5216 (12)
O3—C9	1.4498 (10)	C13—C14	1.5145 (12)
O4—C11	1.3829 (11)	C13—H13A	0.9700
O4—C10	1.4298 (10)	C13—H13B	0.9700
O5—C10	1.3581 (10)	C14—C19	1.3967 (12)
O5—C21	1.4495 (11)	C14—C15	1.3971 (13)
N1—C2	1.3942 (12)	C15—C16	1.3934 (13)
N1—C1	1.3959 (11)	C15—H15A	0.9300
N1—C20	1.4694 (12)	C16—C17	1.3924 (14)
N2—C11	1.2759 (12)	C16—H16A	0.9300
N2—C12	1.4557 (11)	C17—C18	1.3876 (15)
C1—C9	1.5244 (12)	C17—H17A	0.9300
C2—C3	1.4815 (13)	C18—C19	1.3957 (13)
C3—C4	1.3976 (12)	C18—H18A	0.9300
C3—C8	1.3975 (12)	C19—H19A	0.9300
C4—C5	1.3864 (14)	C20—H20A	0.9600
C4—H4A	0.9300	C20—H20B	0.9600
C5—C6	1.3919 (15)	C20—H20C	0.9600
C5—H5A	0.9300	C21—H21A	0.9600
C6—C7	1.3919 (13)	C21—H21B	0.9600
C6—H6A	0.9300	C21—H21C	0.9600
C7—C8	1.3928 (12)	C22—H22A	0.9600
C7—H7A	0.9300	C22—H22B	0.9600
C8—C9	1.4935 (12)	C22—H22C	0.9600
C9—C12	1.5995 (12)		
			
C10—O3—C9	92.84 (6)	N2—C12—C10	104.34 (7)
C11—O4—C10	104.75 (7)	C13—C12—C10	123.07 (7)
C10—O5—C21	114.13 (7)	N2—C12—C9	112.11 (7)
C2—N1—C1	124.06 (8)	C13—C12—C9	117.14 (7)
C2—N1—C20	116.42 (8)	C10—C12—C9	83.10 (6)
C1—N1—C20	118.92 (8)	C14—C13—C12	114.31 (7)
C11—N2—C12	106.97 (7)	C14—C13—H13A	108.7
O1—C1—N1	122.16 (8)	C12—C13—H13A	108.7
O1—C1—C9	122.60 (8)	C14—C13—H13B	108.7
N1—C1—C9	115.08 (7)	C12—C13—H13B	108.7
O2—C2—N1	119.79 (8)	H13A—C13—H13B	107.6
O2—C2—C3	122.87 (8)	C19—C14—C15	118.45 (8)
N1—C2—C3	117.20 (8)	C19—C14—C13	119.99 (8)
C4—C3—C8	120.09 (8)	C15—C14—C13	121.55 (8)
C4—C3—C2	118.95 (8)	C16—C15—C14	120.75 (9)
C8—C3—C2	120.84 (8)	C16—C15—H15A	119.6
C5—C4—C3	120.06 (9)	C14—C15—H15A	119.6
C5—C4—H4A	120.0	C17—C16—C15	120.27 (9)
C3—C4—H4A	120.0	C17—C16—H16A	119.9
C4—C5—C6	119.77 (9)	C15—C16—H16A	119.9
C4—C5—H5A	120.1	C18—C17—C16	119.48 (9)
C6—C5—H5A	120.1	C18—C17—H17A	120.3
C5—C6—C7	120.50 (9)	C16—C17—H17A	120.3
C5—C6—H6A	119.7	C17—C18—C19	120.21 (9)
C7—C6—H6A	119.7	C17—C18—H18A	119.9
C6—C7—C8	119.90 (9)	C19—C18—H18A	119.9
C6—C7—H7A	120.0	C18—C19—C14	120.83 (9)
C8—C7—H7A	120.0	C18—C19—H19A	119.6
C7—C8—C3	119.62 (8)	C14—C19—H19A	119.6
C7—C8—C9	121.81 (8)	N1—C20—H20A	109.5
C3—C8—C9	118.46 (8)	N1—C20—H20B	109.5
O3—C9—C8	113.09 (7)	H20A—C20—H20B	109.5
O3—C9—C1	111.06 (7)	N1—C20—H20C	109.5
C8—C9—C1	113.09 (7)	H20A—C20—H20C	109.5
O3—C9—C12	90.45 (6)	H20B—C20—H20C	109.5
C8—C9—C12	115.70 (7)	O5—C21—H21A	109.5
C1—C9—C12	111.46 (7)	O5—C21—H21B	109.5
O5—C10—O3	113.99 (7)	H21A—C21—H21B	109.5
O5—C10—O4	111.38 (7)	O5—C21—H21C	109.5
O3—C10—O4	110.66 (7)	H21A—C21—H21C	109.5
O5—C10—C12	120.51 (7)	H21B—C21—H21C	109.5
O3—C10—C12	93.50 (6)	C11—C22—H22A	109.5
O4—C10—C12	105.31 (6)	C11—C22—H22B	109.5
N2—C11—O4	118.40 (8)	H22A—C22—H22B	109.5
N2—C11—C22	126.30 (8)	C11—C22—H22C	109.5
O4—C11—C22	115.29 (8)	H22A—C22—H22C	109.5
N2—C12—C13	113.35 (7)	H22B—C22—H22C	109.5
			
C2—N1—C1—O1	161.43 (9)	C9—O3—C10—C12	−2.69 (6)
C20—N1—C1—O1	−9.42 (13)	C11—O4—C10—O5	−136.88 (7)
C2—N1—C1—C9	−23.15 (12)	C11—O4—C10—O3	95.23 (8)
C20—N1—C1—C9	166.00 (8)	C11—O4—C10—C12	−4.62 (8)
C1—N1—C2—O2	−176.58 (8)	C12—N2—C11—O4	−1.64 (11)
C20—N1—C2—O2	−5.52 (13)	C12—N2—C11—C22	178.08 (8)
C1—N1—C2—C3	−0.79 (13)	C10—O4—C11—N2	4.24 (10)
C20—N1—C2—C3	170.27 (8)	C10—O4—C11—C22	−175.51 (7)
O2—C2—C3—C4	7.97 (14)	C11—N2—C12—C13	134.85 (8)
N1—C2—C3—C4	−167.68 (8)	C11—N2—C12—C10	−1.51 (9)
O2—C2—C3—C8	−175.94 (9)	C11—N2—C12—C9	−89.79 (8)
N1—C2—C3—C8	8.41 (13)	O5—C10—C12—N2	130.74 (8)
C8—C3—C4—C5	−2.44 (14)	O3—C10—C12—N2	−108.67 (7)
C2—C3—C4—C5	173.68 (9)	O4—C10—C12—N2	3.87 (9)
C3—C4—C5—C6	0.93 (14)	O5—C10—C12—C13	−0.15 (12)
C4—C5—C6—C7	1.28 (15)	O3—C10—C12—C13	120.45 (8)
C5—C6—C7—C8	−1.96 (14)	O4—C10—C12—C13	−127.02 (8)
C6—C7—C8—C3	0.44 (14)	O5—C10—C12—C9	−118.14 (8)
C6—C7—C8—C9	176.53 (8)	O3—C10—C12—C9	2.45 (6)
C4—C3—C8—C7	1.75 (13)	O4—C10—C12—C9	114.99 (7)
C2—C3—C8—C7	−174.30 (8)	O3—C9—C12—N2	100.30 (7)
C4—C3—C8—C9	−174.47 (8)	C8—C9—C12—N2	−143.66 (8)
C2—C3—C8—C9	9.48 (12)	C1—C9—C12—N2	−12.61 (10)
C10—O3—C9—C8	−115.75 (7)	O3—C9—C12—C13	−126.16 (8)
C10—O3—C9—C1	115.86 (7)	C8—C9—C12—C13	−10.12 (11)
C10—O3—C9—C12	2.59 (6)	C1—C9—C12—C13	120.93 (8)
C7—C8—C9—O3	24.17 (12)	O3—C9—C12—C10	−2.41 (6)
C3—C8—C9—O3	−159.69 (8)	C8—C9—C12—C10	113.63 (8)
C7—C8—C9—C1	151.50 (8)	C1—C9—C12—C10	−115.32 (7)
C3—C8—C9—C1	−32.36 (11)	N2—C12—C13—C14	−42.62 (10)
C7—C8—C9—C12	−78.22 (10)	C10—C12—C13—C14	84.47 (10)
C3—C8—C9—C12	97.91 (9)	C9—C12—C13—C14	−175.60 (7)
O1—C1—C9—O3	−17.55 (12)	C12—C13—C14—C19	126.06 (9)
N1—C1—C9—O3	167.04 (7)	C12—C13—C14—C15	−55.14 (11)
O1—C1—C9—C8	−145.95 (9)	C19—C14—C15—C16	0.68 (13)
N1—C1—C9—C8	38.65 (10)	C13—C14—C15—C16	−178.14 (8)
O1—C1—C9—C12	81.68 (11)	C14—C15—C16—C17	0.27 (14)
N1—C1—C9—C12	−93.73 (9)	C15—C16—C17—C18	−0.75 (14)
C21—O5—C10—O3	55.72 (9)	C16—C17—C18—C19	0.27 (14)
C21—O5—C10—O4	−70.36 (9)	C17—C18—C19—C14	0.70 (15)
C21—O5—C10—C12	165.60 (7)	C15—C14—C19—C18	−1.16 (14)
C9—O3—C10—O5	123.05 (7)	C13—C14—C19—C18	177.68 (8)
C9—O3—C10—O4	−110.49 (7)		

### Hydrogen-bond geometry (Å, °)

**Table:**

*D*—H···*A*	*D*—H	H···*A*	*D*···*A*	*D*—H···*A*
C15—H15A···O5	0.93	2.51	3.3026 (12)	143
C20—H20C···O2^i^	0.96	2.49	3.4424 (13)	174
C21—H21B···N2^ii^	0.96	2.52	3.4018 (12)	152
C22—H22C···O2^ii^	0.96	2.50	3.3854 (12)	154

Symmetry codes: (i) −*x*+2, −*y*+2, −*z*+2; (ii) −*x*+1, *y*−1/2, −*z*+3/2.
